# Gender and Age-Related Trends in Inhalant Allergen Sensitization in Lithuania: A Cross-Sectional Study

**DOI:** 10.3390/ijms26199719

**Published:** 2025-10-06

**Authors:** Gabija Didžiokaitė, Aida Kuznecovaitė, Gabija Biliūtė, Violeta Kvedarienė

**Affiliations:** 1 Faculty of Medicine, Vilnius University, 03101 Vilnius, Lithuania; 2 Faculty of Medicine, Institute of Biomedical Sciences, Department of Pathology, Vilnius University, 03101 Vilnius, Lithuania; 3 Innovative Allergology Center, 06256 Vilnius, Lithuania

**Keywords:** inhalant allergens, allergen sensitization, molecular allergy diagnostics, age-related trends, sex differences, ALEX2 macroarray, respiratory allergy, epidemiology

## Abstract

Sensitization to inhalant allergens is a major factor in the development of allergic diseases. Despite this, few studies have comprehensively analyzed age- and sex-specific patterns within defined populations. This study aimed to investigate the prevalence and distribution of sensitization to inhalant allergens in different demographic groups of the Lithuanian population using molecular diagnostics. We retrospectively reviewed molecular allergy profiles of 658 patients tested with the ALEX2 macroarray between 2020 and 2022. Sensitization to inhalant allergen components was assessed and compared across three age groups (<18, 18–44, >44 years) and by sex. Sensitization to at least one inhalant allergen was observed in 62.16% of patients. Rates were significantly higher in males compared to females, particularly in the reproductive-age group (*p* = 0.0167). Children exhibited the highest prevalence, which declined with age. Tree pollen, pet dander, grass pollen, and dust mites were the dominant allergen groups. Boys were more often sensitized than men, and girls more often than women. Male patients showed higher sensitization to most allergens, except dust mites and weeds in certain female subgroups. Distinct age- and sex-related differences in sensitization patterns were identified. These results emphasize the importance of demographic factors in allergy diagnostics and highlight the need for region-specific sensitization data to inform clinical care and public health strategies.

## 1. Introduction

Inhalant allergens such as pollen, dust mites, mold spores, and animal dander can trigger allergic reactions when inhaled [1]. They play a crucial role in allergic diseases like asthma, allergic rhinitis (AR), and allergic conjunctivitis by provoking the immune system to overreact, leading to symptoms such as sneezing, itching, and respiratory issues [1,2]. Sensitization to these allergens has a significant global impact, with its prevalence increasing due to factors such as urbanization, pollution, and lifestyle changes [3,4,5]. AR alone affects 20% to 30% of adults and up to 40% of children yet remains frequently misdiagnosed and inadequately managed [6,7].

Sensitization patterns are not static; they can change across different life stages. In childhood, boys tend to exhibit higher sensitization rates than girls, possibly due to hormonal and immunological differences. Interestingly, this trend often reverses in adulthood, with females becoming more sensitized, driven by hormonal shifts, environmental exposures, and lifestyle factors [8,9,10]. Understanding how sensitization evolves from childhood through adulthood and into older age is crucial for tailoring medical interventions and improving patient outcomes. This evolution reflects a complex interplay between genetic predispositions, environmental exposures, and physiological changes over time [11,12,13].

Despite extensive research, few studies jointly examine age- and sex-related sensitization trends within defined populations, including Lithuania. Detailed research into these trends is essential for understanding how allergic sensitization manifests in diverse groups and in specific geographic areas with its typical flora and environmental factors. For instance, investigating populations such as Lithuanian inhabitants can provide valuable insights into local variations in sensitization patterns. Such studies are necessary to fill knowledge gaps and inform targeted public health strategies.

This study aims to analyze gender- and age-related trends of inhalant allergen sensitization in Lithuanian and compare them. By addressing the gap in localized data, this study seeks to contribute valuable insights to the global understanding of allergic diseases.

## 2. Results

A total of 658 patients were included in the study, with a median age of 21 years (IQR: 30). The age ranged from 0.2 to 75 years. Females represented 51.22%, males—48.78%. By age, children < 18 years accounted for 47.11% (44.52% girls, 55.48% boys), adults of reproductive age (18–44 years) 42.86% (54.26% female, 45.74% male), and those over 44 years 10.03% (69.70% female, 30.30% male) (Table 1).

### 2.1. Sensitization Profile of the Sample Group and Sex-Dependent Differences

Overall, 409 (62.16%) were sensitized to ≥1 inhalant allergen, with males more frequently sensitized than females (*p* = 0.0199). A comparable percentage of children and adults were found to be sensitized: 197 children (63.55%) vs. 212 adults (60.13%). There were no statistically significant differences in overall sensitization between children and adults (*p* = 0.4877), and the prevalence of sensitization did not differ significantly between males and females in the children group (*p* = 0.6870).

When the female subgroup was analyzed separately, the highest sensitization rates were observed in girls (62.32%), while the lowest rates were found in females over 44 years old (54.35%). Conversely, in the male group, males aged 18–44 showed the highest rates of sensitivity (69.23%). Boys had the lowest sensitization rates among males (64.53%).

Overall, males across all age groups were established to be more frequently sensitized than females, although this difference was not always statistically significant due to the relatively small sample sizes in some age groups (Table 2). In the subgroup of individuals of reproductive age, a statistically significant difference was found between the sexes. Males in this age group were found to be sensitized to at least one allergen component more frequently than females, with a *p*-value of 0.0167.

**Figure 1:**
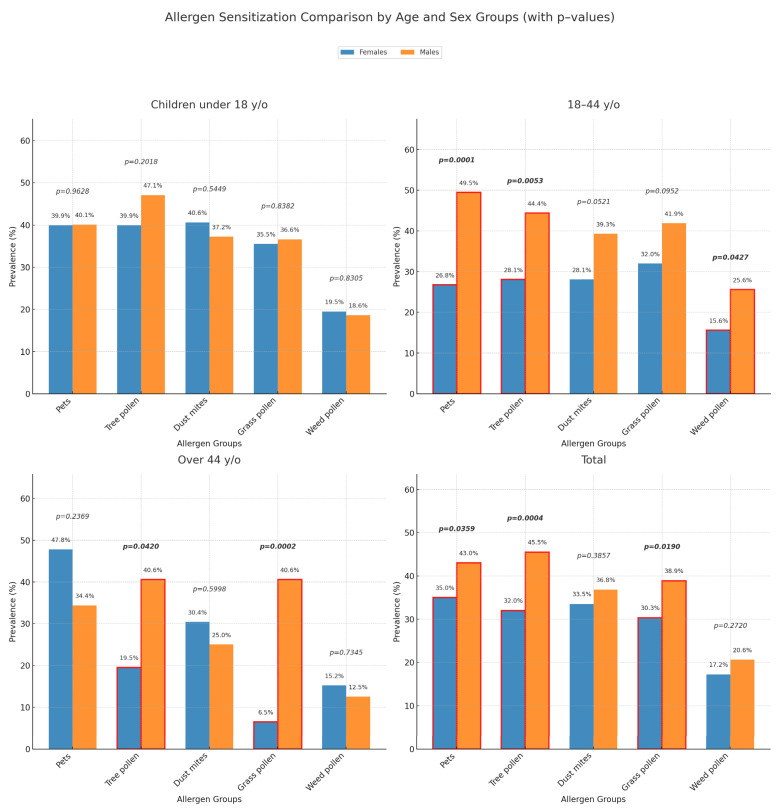
The differences between females and males in age subgroups for the most common sensitizing allergen groups. The red rectangular frame represents the differences which were statistically significant.

Sensitization to pets was the most common overall—256 (38.9%) patients—followed by tree pollen (38.6%), dust mites (35.1%), and grass pollen (34.5%). More than one-third of all patients were found to be sensitized to at least one allergen from these allergen groups. For females, in general, sensitization to pets was the most frequent (35.0%), while for males, the most frequent sensitization was diagnosed to tree pollen (45.5%). When patients were analyzed separately according to age groups, the most frequent sensitizing allergen group for each age group differed. However, in most age groups for both genders, the four most common sensitizing allergen groups remained pets, tree pollen, dust mites, and grass pollen. The only exception was females over 44 years old, whose rates of sensitization to grass pollen decreased significantly compared to younger female groups. In this age group, only 8.7% of females were sensitized to grass pollen. Consequently, sensitization to weed pollen rose to become one of the four most common allergen groups (Figure 1).

### 2.2. Sensitization Differences for the Most Common Inhalant Allergen Groups Between Subgroups of Different Age and Sex

Children most often were sensitized to tree pollen, affecting 136 (43.9%) individuals in this age group. More than one-third were sensitized to pets (n = 124; 40.0%), dust mites (n = 120; 38.7%), and grass pollen (n = 112; 36.1%). Children were also found to be significantly more frequently sensitized to tree pollen (*p* = 0.009) and animals (*p* = 0.009) compared to adults (both those of reproductive age and those older than 44).

Among patients of reproductive age, sensitization to pets and grass pollen was the most prevalent, with 99 (36.7%) and 98 (36.3%) patients affected, respectively. In patients older than 44 years, the most common sensitization was to pets, observed in 33 (42.3%) patients (Figure 1).

When comparing by sex across different age, significant differences were found between females and males. Men were more often sensitized overall to pets (*p* = 0.0359), trees (*p* = 0.0004), and grasses (*p* = 0.019). Among individuals of reproductive age, men were frequently sensitized to pets (*p* = 0.0001), tree pollen (*p* = 0.0053), and weed pollen (*p* = 0.0427). Women over 44 years old were statistically significantly less frequently sensitized to tree pollen (*p* = 0.042) and grass pollen (*p* = 0.0002) compared to men in the same age group (Figure 1). Interestingly, no statistically significant differences were observed between children of male and female sex regarding sensitization to the most common allergen groups (Figure 1).

### 2.3. Sensitizations to the Most Common Sensitizing Allergen Components in Allergen Groups

In general, the most common sensitizing inhalant allergens for the whole sample group were silver birch allergen Bet v 1 (33.6%), cat’s allergen (uteroglobin) Fel d 1 (31.2%), timothy grass allergen Phl p 1 (26.1%), and Dermatophagoides farinae dust mite allergen Der f 2 (23.6%).

Among grass allergens, the most frequent sensitizing allergen components for all patients were beta-expansin timothy grass allergen Phl p 1 (26.1%), serving as the primary sensitizer, followed by Phl p 5.0101 (14.2%). Cross-reactions were also observed with other group 1 grass allergens, including perennial ryegrass allergen Lol p 1 (23.3%), bermuda grass Cyn d 1 (16.3%), and cultivated rye allergen Sec c pollen (17.9%).

Within tree pollen allergens, the sample group was mostly sensitized to three PR-10 allergens. Silver birch allergen Bet v 1 (33.6%) was identified as the primary sensitizer, with cross-reactions observed to beech allergen Fag s 1 (27.4%), hazel pollen allergens Cor a 1.0103 (26.0%), Cor a pollen (22.3%), and alder allergen Aln g 1 (22.8%).

For dust mites, the most frequent sensitizing components were NPC2 family members, Dermatophagoides farinae allergen Der f 2 (23.6%) and Dermatophagoides pteronyssinus Der p 2 (23.3%). Additionally, cysteine protease family members of Dermatophagoides pteronyssinus allergen, Der p 1 (18.8%), and Dermatophagoides farinae, Der f 1 (18.5%), were significant, along with the peritrophin-like protein Dermatophagoides pteronyssinus allergen Der p 23 (18.2%).

In the pet allergens group, the most common sensitizing components were cat allergen (uteroglobin) Fel d 1 (31.2%), male dog urine allergen Can f 5 (17.5%), dog allergen (lipocalin) Can f 1 (15.5%), and cat allergen (lipocalin) Fel d 7 (14.4%).

**Figure 2:**
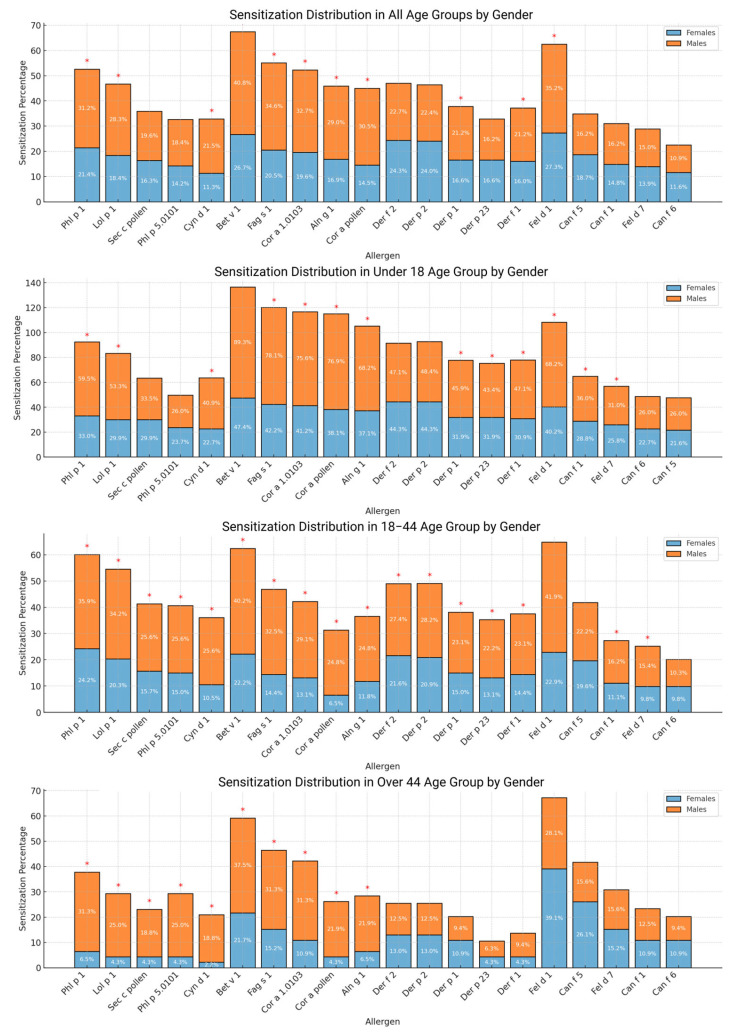
The stacked bar charts illustrate the distribution of allergen sensitization percentages among males and females across various allergens and age groups. Each chart corresponds to a specific age group, with bars representing individual allergens. The lower segment of each bar shows the percentage of females sensitized, while the upper segment indicates the percentage of males. Notably, red asterisks (*) indicate allergens with significant gender differences in sensitization rates.

When analyzing the data separately by sex, the most frequently sensitizing allergen components remained consistent for both genders across almost all age subgroups. However, in the children’s group, boys were most commonly sensitized to Der f 1 and Der p 1, whereas girls, along with subjects of both sexes in other age groups, were most frequently sensitized to Der f 2 and Der p 2. In addition, males across all age groups were more often sensitized to most inhalant allergen components compared to females. The exceptions to this trend were observed among females over 44 years old, who showed higher sensitization rates to dust mites and pet allergens. Overall, dust mite sensitization was also more prevalent among females (Figure 2).

### 2.4. Sensitization Differences for Allergen Components Between Subgroups According to Sex

When sensitizations to specific allergens were compared between sexes, additional statistically significant differences were discovered. Males were observed to be statistically significantly more frequently sensitized to eight allergen components of the grass pollen allergen group in comparison with females: Phl p 1, Lol p 1, Cyn d 1, Cyn d, Pas n, Phl p 2, Phl p 6, and Phl p 12. Also, males were found to be statistically significantly more frequently sensitized to eight tree pollen allergen components in comparison with females: Bet v 1, Fag s 1, Cor a 1.0103, Aln g 1, Cor a pollen, Jur g pollen, Pho d 2, and Pla a 3. Finally, males were statistically significantly more frequently sensitized to three weed allergens in comparison with females: Ama r, Mer a 1, Sal k 1; and to cat allergen component Fel d 1: 92 (27.3%) vs. 113 (35.2%), *p* = 0.029. Interestingly, when sensitization rates were compared between male and female subgroups for dust mites allergens, no statistically significant differences were discovered.

### 2.5. Sensitization Profiles of the Age Subgroup Under 18 Years Old

In children, the most common allergen components were identified across various allergen groups. For tree pollen, the predominant allergens were silver birch allergen Bet v 1 (38.1%), beech allergen Fag s 1 (33.2%), hazel pollen allergens Cor a 1.0103 (32.9%), and alder allergen Aln g 1 (29.4%). Within the grass pollen group, the primary sensitizing agent was timothy grass allergen Phl p 1 (25.8%), with frequent cross-reactions to perennial ryegrass allergen Lol p 1 (23.2%), cultivated rye allergen Sec c pollen (18.0%), and bermuda grass Cyn d 1 (17.7%).

Dust mites were another frequent source of sensitization in children, particularly allergens from the NPC2 family, including Dermatophagoides farinae allergen Der f 2 (25.8%) and Dermatophagoides pteronyssinus allergen Der p 2 (25.2%), as well as cysteine protease family members, such as Dermatophagoides pteronyssinus allergen Der p 1 (22.6%) and Dermatophagoides farinae allergen Der f 1 (21.9%), along with the peritrophin-like protein Dermatophagoides pteronyssinus allergen Der p 23 (21.3%). Among the pet allergens, cat allergen Fel d 1 (30.3%) was the most common, followed by dog allergen Can f 1 (19.4%) and male dog urine allergen Can f 5 (13.5%).

In the children‘s group, statistically significant differences were observed between girls and boys in sensitization to tree pollen allergen of Mediterranean cypress Cup s; weed pollen allergens: white goosefoot allergen Che a, saltwort allergen Sal k 1, and common nettle allergen Urt d; dust mite allergens: Dermatophagoides pteronyssinus allergen Der p 2 and Blomia tropicalis allergen Blo t 10; and pet allergen of European rabbit Ory c 3. Girls were found to be statistically significantly more frequently sensitized to tree pollen allergen Cup s (4.3% vs. 0.0%, *p* = 0.006); weed pollen allergens Che a (2.9% vs. 0.0%, *p* = 0.038) and Urt d (2.9% vs. 0.0%, *p* = 0.038); dust mite allergen Der p 2 (31.2% vs. 20.3%, *p* = 0.029); and pet allergen Ory c 3 (10.1% vs. 3.5%, *p* = 0.018) in comparison with boys, while boys were found to be statistically significantly more frequently sensitized to weed pollen allergen Sal k 1 (0.0% vs. 4.1%, *p* = 0.015) and dust mite allergen Blo t 10 (0.0% vs. 2.9%, *p* = 0.043) in comparison with girls of the same age subgroup.

### 2.6. The Most Common Sensitizing Allergen Components for Age Subgroup 18–44 Years Old and Sensitization Differences According to Sex

For patients aged 18–44 years, similar allergen patterns emerged across different allergen groups. In the tree pollen group, the most common sensitizing allergen components were silver birch allergen Bet v 1 (30.0%), beech allergen Fag s 1 (22.2%), hazel pollen allergen Cor a 1.0103 (20.0%), and alder allergen Aln g 1 (17.4%).

In the grass pollen group, timothy grass allergen Phl p 1 (29.3%), perennial ryegrass allergen Lol p 1 (26.3%), cultivated rye allergen Sec c pollen (20.0%), and bermuda grass Cyn d 1 (16.7%) were the most prevalent.

For house dust mites, the most frequent allergens were NPC2 family members Dermatophagoides farinae allergen Der f 2 (24.1%) and Dermatophagoides pteronyssinus allergen Der p 2 (24.1%), with additional sensitization observed to cysteine protease family members Dermatophagoides pteronyssinus Der p 1 (18.1%) and Dermatophagoides farinae Der f 1 (18.1%). The peritrophin-like protein Dermatophagoides pteronyssinus allergen Der p 23 (17.8%) was also significant.

In the pet allergen group, the most common sensitizing agents were cat allergen (uteroglobin) Fel d 1 (31.1%), male dog urine allergen Can f 5 (20.7%), dog allergen (lipocalin) Can f 1 (13.3%), and cat allergen (lipocalin) Fel d 7 (12.2%).

When subgroup of individuals of reproductive age was analyzed separately according to sex, statistically significant differences were observed between females and males in sensitization to grass pollen, tree pollen, weed pollen, dust mites, pets, yeast, and mold allergen groups. The males of reproductive age were statistically significantly more frequently sensitized to grass pollen allergens Cyn d, Cyn d 1, Lol p 1, Pas n, Phl p 1, Phl p 5.0101, Phl p 6, Phl p 12, Phr c, and Sec c pollen; tree pollen allergens Aln g 1, Bet v 1, Cor a 1.0103, Cor a pollen, Fag s 1, and Pho d 2; weed pollen allergens Ama r, Can s 3, Che a, Mer a 1, and Sal k; dust mite allergen Der p 23, and cat allergen Fel d 1. Females of reproductive age were statistically significantly more frequently sensitized only to one weed pollen allergen Amb a 4.

### 2.7. The Differences in Sensitization Patterns to Inhalant Allergens of Males and Females Between All Adults of the Sample

When the data of adult patients both, of reproductive age and older, were analyzed, adult males were found to be statistically significantly more frequently sensitized to a majority of grass allergen components in comparison with females: Cyn d (*p* < 0.001), Cyn d 1 (*p* < 0.001), Lol p 1 (*p* < 0.001), Pas n (*p* < 0.001), Phl p 1 (*p* = 0.002), Phl p 2 (*p* = 0.005), Phl p 5.0101 (*p* = 0.002), Phl p 6 (*p* = 0.003), Phl p 12 (*p* = 0.022), Phr c (*p* = 0.003), and Sec c pollen (*p* = 0.007). Also, adult males were statistically significantly more frequently sensitized to seven tree pollen allergen components: Aln g 1 (*p* < 0.001), Bet v 1 (*p* < 0.001), Cor a 1.0103 (*p* < 0.001), Cor a pollen (*p* < 0.001), Fag s 1 (*p* < 0.001), Pho d 2 (*p* = 0.32), and Pla a 3 (*p* = 0.043). Also, adult males were statistically significantly more sensitized to Der f 1 (*p* = 0.04) and Fel d 1 (*p* = 0.015) in comparison with females. Also, none of the adults of both sexes were sensitized to Bro p, Mor r, and Ole e 9 allergens.

### 2.8. The Most Common Sensitizing Allergen Components for Age Subgroup over 44 Years Old

In individuals over 44 years old, sensitization to the tree pollen group also featured prominently, with the highest sensitization to silver birch allergen Bet v 1 (28.2%), beech allergen Fag s 1 (21.8%), hazel pollen allergen Cor a 1.0103 (19.2%), and alder allergen Aln g 1 (15.4%).

In grass pollen group, the most common sensitizing allergen components were timothy grass allergen Phl p 1 (16.7%), perennial ryegrass allergen Lol p 1 (12.8%), timothy grass allergen Phl p 5.0101 (12.8%), and cultivated rye allergen Sec c pollen (10.3%).

In the house dust mite allergen group, NPC2 family members Dermatophagoides farinae allergen Der f 2 (12.8%) and Dermatophagoides pteronyssinus allergen Der p 2 (12.8%) were the most common, with lower sensitization to peritrophin-like protein Dermatophagoides pteronyssinus allergen Der p 23 (7.7%) and cysteine protease family members Dermatophagoides pteronyssinus Der p 1 (6.4%) and Dermatophagoides farinae Der f 1 (6.4%).

In pet allergen components group, the highest sensitization rates were observed for cat allergen Fel d 1 (34.6%), male dog urine allergen Can f 5 (21.8%), and cat allergen Fel d 7–12 (15.4%).

When the sensitization profile of patients older than 44 years old was analyzed and compared between sexes, statistically significant differences were observed between females and males in the grass pollen, tree pollen, and weed pollen allergen groups. However, no statistically significant differences were observed for dust mite and pet allergen components.

Males over 44 years old, in comparison with women, were statistically significantly more frequently sensitized to grass pollen allergens Cyn d 1, Cyn d, Pas n, Phl p 1, Phl p 2, Phl p 5.0101, Phl p 6, and Sec c pollen; tree pollen allergens Aln g 1, Cor a 1.0103, Cor a pollen, and Jug r pollen; and weed pollen allergen Amb a 4. Females over 44 years old were not statistically significantly more frequently sensitized to any of the inhalant allergens available in the ALEX2 macroarray in comparison with males.

### 2.9. Differences in Sensitization to Inhalant Allergen Components Between Children and Adults

When comparing children to adults (both of reproductive age and older), children were statistically significantly more frequently sensitized to a broad range of inhalant allergens. Notably, children were more frequently sensitized to tree pollen allergens such as Phr c (10.0% vs. 5.7%, *p* = 0.042), Aln g 1 (29.4% vs. 17.0%, *p* < 0.001), Bet v 1 (38.0% vs. 29.6%, *p* = 0.022), and Cor a 1.0103 (32.9% vs. 19.8%, *p* < 0.001).

In the grass pollen group, significant differences were also observed, with children showing higher sensitization rates to components such as Bet v 2 (6.5% vs. 2.0%, *p* = 0.004) and Bet v 6 (4.8% vs. 1.7%, *p* = 0.005) in comparison with adults. Sensitization was also more frequent for certain weed pollen allergens, such as hemp allergen Can s (3.9% vs. 0.9%, *p* = 0.01) and annual mercury allergen Mer a 1 (5.5% vs. 1.1%, *p* = 0.002) in the children’s group.

In the dust mite group, children were more frequently sensitized to Der f 1 (21.9% vs. 15.5%, *p* = 0.034) and Der p 1 (22.6% vs. 15.5%, *p* = 0.021) than adults. Sensitization differences were also observed in the pet allergen group, with children being more frequently sensitized to Can f 6 (13.9% vs. 8.9%, *p* = 0.044), Ory c 2 (1.9% vs. 0.3%, *p* = 0.04), and Ory c 3 (6.5% vs. 3.2%, *p* = 0.047). Interestingly, adults were found to be more frequently sensitized to male dog urine allergen Can f 5 (21.0% vs. 13.5%, *p* = 0.012).

### 2.10. Sensitization Differences Between Children and Adults According to Sex

When analyzing sensitization by sex, girls were statistically significantly more frequently sensitized to tree pollen (*p* = 0.011), dust mites (*p* = 0.022), and farm animal allergens (*p* = 0.009) compared to adult women. However, no significant differences were found between boys and adult men for sensitization to any allergen group. In both sexes, children showed a higher sensitization frequency for the majority of tested allergen component groups compared to adults.

Secondly, when sensitizations for specific inhalant allergen components were compared between children and adults separated by sex, girls and boys were found to be more frequently sensitized to the majority of allergen components in comparison with women and men, accordingly. However, only for the hazel pollen allergen Cor a pollen were children found to be statistically significantly more frequently sensitized than adults in both, female and male groups—sensitization differences to other allergen components between children and adults were only significant either in the male or the female group. Interestingly, sensitization to common nettle allergen Urt d was statistically significantly higher for girls in the female group (*p* = 0.027), while in the male group, it was found statistically significantly more frequently in adult males in comparison with boys (*p* = 0.045). For other allergen components, statistically significant differences in sensitization for children and adults were found only in the female or only in the male group, and children were sensitized more frequently than adults in all these cases.

## 3. Discussion

Sensitization to inhalant allergens is an important factor in diagnosis and management of respiratory allergic diseases. Understanding the patterns of allergen sensitization across different demographics helps to address the growing global burden of allergic conditions. In our study, we analyzed the age- and sex-specific sensitization patterns in Lithuania using molecular profiles. Evidence on how sensitization varies across the lifespan, especially by sex, remains limited. However, recent research has shed light on the variations in the prevalence of different allergic diseases across genders and among different age groups within the general population. Our findings reveal sex- and age-related differences, supporting the role of biological, environmental, and hormonal influences on sensitization.

### 3.1. Prevalence of Sensitization to Inhalant Allergens in Different Populations

Our study revealed that 62.16% of the Lithuanian patients were sensitized to ≥1 inhalant allergen, underscoring a substantial public-health burden. Comparable rates are reported worldwide: A total of 44.6% individuals aged ≥6 years in the United States, according to National Health and Nutrition Examination Survey, showed sensitization to inhalant allergens in 2005–2006 [14], 41.97% among suspected allergy patients in China’s Henan Province [15], and 45.3% in a South Korean multicenter study [16]. In Europe the prevalence of sensitization to inhalant allergens was observed to be slightly lower, yet still very alerting, with 40.2% of children and adolescents in Germany [17] and 37.7% of the population in Wroclaw, Poland, sensitized to at least one inhalant allergen [18]. The data from other European countries in sensitization patterns and rates is still missing and may have some significant differences due to different geographic and weather conditions, industrialization levels, and other factors important for the development of sensitizations and allergic diseases.

### 3.2. Most Common Allergen Groups in Different Populations

In Lithuanian, sensitization was highest to pets (38.9%), then tree pollen (38.6%), dust mites (35.1%), and grass pollen (34.5%). Similar profiles are reported elsewhere, with house dust mites, pollens, and molds predominating in Germany and South Korea [16,17]. Across Europe, patterns vary by country: in three areas of Sweden, birch, grass, cat, and dog allergens were most common (each around 15%) [19]. In Germany, among children and adolescents, timothy grass and rye pollen allergen components were most prevalent (22.7% and 21.2%) [17,20]. In our study, sensitization was analyzed to six allergen components of the timothy grass, and the sensitization to these components within children and adolescents in our sample was distributed accordingly: Phl p 1—25.8%, Phl p 2—10.0%, Phl p 5.0101—14.2%, Phl p 6—10.6, Phl p 7—2.3, and Phl p 12—5.8. We also tested for the rye pollen allergen component Sec c pollen and found the sensitization prevalence of 18.1%.

Globally, both similarities and differences emerge when comparing Europe to other regions. In Henan Province, China, by the study carried out between 2012 and 2016, the most common inhalant allergens were determined to be allergen components of *Dermatophagoides pteronyssimus*, cockroach, and mold mix, with sensitization rates of 22.79%, 9.00%, and 8.38%, respectively [15]. However, information regarding sensitization to specific allergen components is lacking. In Indonesia, Jakarta, high sensitization rates were observed for house dust mites *Dermatophagoides pteronyssinus* (77.57%) and *Blomia tropicalis* (71.96%) among allergic patients [21]. In South Korea, the most common inhalant allergens, according to a study published in 2017, were *Dermatophagoides farinae* and *Dermatophagoides pteronyssinus*, with regional variations in allergen sensitization patterns [16]. These findings emphasize the complex interplay of environmental and genetic factors that shape allergen sensitization patterns worldwide.

### 3.3. Geographical Differences as a Factor Influencing Different Sensitization Patterns to Inhalant Allergens in Different Populations

Sensitization patterns vary with geography not only due to a different local allergen exposure of distinct flora and fauna characteristics to respective territories, but also due to different climate zones, humidity levels, environmental factors, and urbanization levels that may shape these variations. For example, according to Song et al., urbanization is associated with higher sensitization rates; in South Korea, it was observed that urban areas show greater sensitization to house dust mites and increased rhinitis symptoms compared to rural areas [22]. Moreover, Toth et al. found that individuals living in urban areas of Zagreb, Croatia, were more often sensitized to birch, whereas rural residents showed more ambrosia sensitization [23]. Climatic conditions also play a significant role. In France, children living in coastal cities like Marseille and Bordeaux had a higher prevalence of allergy to indoor allergens such as house dust mites (HDM) and cat dander but lower birch sensitization likely due to higher humidity [24]. These trends highlight that regional climates may contribute to the observed high prevalence of pollen and dust mite sensitization when specific environmental factors such as urbanization, temperature, and humidity interact. On the contrary, in subtropical and tropical regions, sensitization to HDM is notably higher due to favorable conditions for their growth. The increase in HDM-related allergies is less clearly linked to environmental changes compared to pollen allergens, but it remains significant [25]. Though Lithuania’s temperate climate has marked seasons, the higher prevalence of HDM sensitization in our study may suggest that indoor environmental factors, such as home humidity levels or heating systems, may mimic similar effects. More studies are imperative to further analyze the impact of weather and other geographic conditions on sensitization patterns. Additional studies using molecular diagnostics are imperative for better understanding of the occurring sensitization patterns in different geographic locations.

We observed a high general prevalence of sensitization to male dog urine allergen component Can f 5 across all age groups, with statistically significantly higher rates in adults than in children. Clinically, this should prompt consideration of the rare human seminal plasma allergy, which is often misdiagnosed for vulvar infections and is not addressed properly for women [26,27]. This allergy may develop if a woman is sensitized to Can f 5 and is not managed sufficiently, as a cross-reaction with prostate-specific allergen (PSA) may occur, leading not only to physical discomfort when in contact with male semen, but also as a potential cause of women infertility [27].

### 3.4. Age-Related Differences

Age plays a significant role in shaping sensitization patterns to inhalant allergens, with distinct trends observed across different life stages. In children, sensitization to aeroallergens such as HDM and pollen increases with age, particularly between ages five to nine [28,29]. Those <5 years are more commonly sensitized to indoor allergens, such as HDM. Studies from Emin et al., Sporik et al., and Niemeijer et al. show that infants and children with asthma often test positive for HDM, with rates higher than for outdoor allergens like pollen [30,31,32]. As children grow, sensitization shifts toward outdoor allergens such as pollen, particularly grass pollen, with a marked increase around 10 to 15 years old [30,33].

Moreover, Fuseini et al. have also underscored the significant differences in asthma prevalence between males and females across various life stages: during childhood (up to age 15), boys tend to exhibit a higher prevalence of asthma (11.9%) compared to girls (7.5%) [34]. Through adolescence and adulthood, rates drop in males (6.2%), but rise in females (10.4%), then decline after menopause [35]. In our study, men showed higher sensitization rates to the majority of inhalant allergens in all age groups—from children to older patients, except for sensitization to dust mites, where girls were sensitized more frequently than boys (*p* = 0.5449), to pet allergens, where women older than 44 years old were sensitized more frequently than men (*p* = 0.2369), and to weed pollen, where girls and women over 44 years old were sensitized more frequently than men (*p* = 0.8305 and *p* = 0.7345, accordingly).

Hong et al. investigated the prevalence of allergen sensitization and allergic rhinitis symptoms among 2883 patients and revealed a notable pattern in allergen sensitization prevalence across age groups. The prevalence of sensitization to allergens increased from ≤6 years through the 20s, then declined from about age 30. Specifically, sensitization to *Dermatophagoides pteronyssinus* and *Dermatophagoides farinae* decreased with age, while sensitization to weeds mugwort and ragweed rose until the age of 70 [36].

According to a study by Barbee et al. published in 1981 and a study by Niemeijer et al. published in 1992, the highest prevalence of sensitization was observed in the 20–34 year age group, with a decline in prevalence as age increases [30,37]. Zhao et al. found that for individuals under 52 years old, sensitization to inhalant allergens, especially HDM, is more prevalent than food allergy [38]. Warm et al. found that prevalence of allergen sensitization decreases with age, except for allergens like grass pollen, which remains significant [39].

These age-related trends align with our Lithuanian data: Children showed higher sensitization rates compared to adults, particularly for tree pollen and dust mites. Among children, sensitization to tree pollen was the most frequent, while HDM sensitization affected more than one third of the subgroup. This pattern reflects the transition from sensitivity to indoor allergens in younger children to increasing sensitivity to outdoor allergens as they grow older.

Interestingly, sensitization rates declined with older age, from 18–44 years (61.11%) to those over 44 years old (60.26%).

### 3.5. Sex-Related Differences

Our Lithuanian findings on sex-related sensitization mirror global trends and reflect a complex interplay of biological, hormonal, and environmental factors. Consistent with prior studies by Ballardini et al. and Melen et al. and the meta-analysis by Goldhahn et al., boys in childhood exhibit higher sensitization rates to airborne allergens, including *Dermatophagoides pteronyssinus* and *Dermatophagoides farinae*, compared to girls [40,41,42]. Bertelsen et al., likewise, found that among children with current rhinitis, boys were more often sensitized to inhalant allergens than girls (83.9% vs. 62.5%) [43]. We observed the same tendency; however, no data on clinical symptoms was available in our study.

Male predominance in childhood sensitization aligns with established global patterns, which suggest that biological differences, such as innate immune response and androgenic effects, may contribute to these observations. For example, according to a study published by Uekert et al., boys exhibit higher cytokine responses, such as IFN-gamma, IL-5, and IL-13, which are associated with increased sensitization and allergic reactions. These responses are more pronounced in boys who wheeze during their third year of life, indicating a stronger innate immune response compared to girls [44].

Pubertal hormonal shifts further reshape sensitization and allergic diseases. There are contradictory data regarding the sensitization patterns for individuals of reproductive age. For example, in a study by Hong et al., higher rates of allergen sensitivity among males compared to females was also observed (48.7% and 44.1%, respectively), which was also observed in our study [36]. However, studies of Dor-Wojnarowska et al. and Goldhahn et al. report female predominance in allergic airway diseases after puberty [18,41]. These patterns are further supported by evidence that estrogen and other sex hormones influence immune responses, potentially amplifying allergic inflammation in females [45,46].

### 3.6. Hormonal Influence on Allergic Diseases

As mentioned above, our study shows sex-related differences to inhalant allergens sensitization across age groups. Current knowledge suggests that hormonal fluctuations and changes in the microbiome throughout a woman’s lifetime can significantly influence allergy rates [47,48]. Moreover, hormonal variations during the menstrual cycle, particularly during ovulation or menstruation, may impact the presentation of allergies [49,50]. The menstrual cycle can also make a difference in the diagnosis of allergy using the skin prick method [51,52]. The increase in skin prick test reactivity at mid-cycle is correlated with elevated levels of estradiol and luteinizing hormone (LH) [51]. While Kirmaz et al.’s study noted that histamine reactivity did not vary significantly throughout the menstrual cycle [51], Kalogeromitros et al. observed an increase in histamine-induced weal-and-flare reactions during the mid-cycle phase [52]. Factors such as early menarches and multiple pregnancies, leading to prolonged exposure to estrogen, have been associated with an elevated risk of developing asthma, including severe cases. These epidemiological findings regarding asthma prevalence across genders have been linked to fluctuations in sex hormone levels within the body, underscoring the pivotal role of sex hormones in the pathogenesis of asthma [34]. Moreover, early studies have reported that 30–40% of females with asthma experience worsening asthma symptoms, decreased peak flow rates, and increased use of rescue medications during the pre- or perimenstrual phase of the menstrual cycle [53,54,55], while Juniper et al., in their study investigating airway responsiveness to methacholine during the menstrual cycle, observed an increase in asthma symptoms during menstruation. Consequently, the mechanisms underlying these cyclic changes in some females remain unclear. However, our study discovered contradictory results regarding female’s sensitization patterns in different periods of their lifetimes. Firstly, on the contrary to the usually observed pattern, which is associated with female hormonal levels, young girls were found to be significantly more frequently sensitized to inhalant allergens than females of reproductive age in our sample. Moreover, females of reproductive age, who are thought to be more sensitized due to the heightened hormonal levels and their frequent fluctuations, were found to be less sensitized than males of the corresponding age group. However, these differences may be partly explained by the specific sample group of the Lithuanian population that was analyzed in our study (and may have some limitations when analyzing its sensitization patterns.)

### 3.7. Limitations

The main limitation of our investigation was the unavailability of patients’ clinical histories, which restricted us from the possibility of raising more complex hypotheses regarding the currently discovered sensitization patterns of different age and sex subgroups. Therefore, our study cannot directly link sensitization prevalence with objectively confirmed allergic disease, and the reported sensitization rate of 62.2% should not be interpreted as the prevalence of clinical Type I hypersensitivity in the population. Moreover, as mentioned previously, the data may not fully represent the prevalence of sensitization of the whole diverse Lithuanian population as our data was collected from the patients who voluntarily referred to the allergist-immunologist. Therefore, the sample group primarily consisted of patients who were sufficiently concerned about their health to voluntarily undergo allergological testing. Thus, our results mainly reflect sensitization patterns in a referred patient cohort and cannot be directly generalized to the overall Lithuanian population. This limitation should be considered when interpreting the findings, as voluntary health-seeking behavior may introduce selection bias.

Also, it is important to note that as this study was a cross-sectional study, it did not follow the same patients over time and only analyzed patients’ sensitization patterns at a single point in time. Therefore, the obtained results can only reveal the sensitization patterns of the current Lithuanian population subgroups of different age and sex as well as to compare them to promote the most significant differences, which would be crucial for further research. However, this study does not have the power to identify the causation of these differences or to predict the changes in sensitization patterns of the same sample group in the future.

Another limitation is the lack of information on patients’ residential areas (urban vs. rural), socioeconomic background, and environmental exposures (e.g., air pollution, indoor humidity, heating systems). These factors are known to strongly influence sensitization patterns, as demonstrated in previous European studies, but were unavailable in our dataset. Therefore, unmeasured confounding from such variables cannot be excluded. However, the results obtained from our study well represent the sensitization profiles and current patterns of the Lithuanian inhabitants from different age and sex subgroups and allow to raise hypotheses and draw conclusions regarding the most common cross-reactivity development patterns for Lithuanian inhabitants. These are invaluable insights for the development of the most suitable healthcare strategies for allergy management in Lithuania and its adaptation to effectively meet the specific needs associated with Lithuanian population.

## 4. Materials and Methods

A retrospective study was conducted using anonymized test results of 658 patients in Lithuania, covering the period from 2020 to 2022. Patients were included based on a suspicion of atopic disease and underwent routine screening for specific immunoglobulin E (sIgE) reactivity using the ALEX2 macroarray (MacroArray Diagnostics GmbH, Vienna, Austria). The ALEX2 macroarray is a multiplex sIgE test, which includes 295 antigens, comprising 117 whole extracts, and 178 molecular components. For group-level analyses (e.g., pets, tree pollen, dust mites, grass pollen, weed pollen), a subject was considered positive for that group if IgE to at least one component within the group exceeded the positivity threshold (≥0.3 kUA/L). Thus, each individual was counted only once per allergen group, regardless of the number of positive components within that group. Patients were considered sensitized if they had confirmed sensitivity to at least one allergen component. In this study, the focus was on the molecular allergy profiles across different age and sex groups.

Patients were categorized into three age groups: children (under 18 years), adults of reproductive age (18–44 years), and those over 44 years. The cut-off at 44 years was chosen because it approximates the later reproductive period in women and the beginning of perimenopausal age, which may influence allergic disease patterns [56,57]. Additionally, epidemiological data suggest that allergen sensitization tends to decline in older adulthood, and this grouping allowed us to capture that transition [58]. While there is no universally accepted cut-off at 44 years, a number of scientific publications have established that women’s reproductive potential, even with the use of assisted reproductive techniques, tremendously decline after 44 years [59,60,61]. Therefore, this threshold was selected to balance biological considerations and ensure adequate subgroup sizes for statistical analysis. Also, the sample was additionally divided into two subgroups based on their gender. Sensitization was defined as a detection of sIgE levels of 0.3 kUA/L or higher for a specific allergen. The study was approved by the Vilnius Regional Biomedical Research Ethics Committee of Vilnius University and conducted in accordance with all ethical requirements.

Statistical analysis was performed using IBM SPSS Statistics 28.0 and RStudio v 4.3.3. Baseline and demographic characteristics were summarized using medians and interquartile ranges for continuous variables and percentages for categorical variables. Differences between groups were analyzed using two-sample Wilcoxon (or Kruskal–Wallis) tests for nonparametric continuous variables and χ^2^ tests for categorical variables. A *p*-value of <0.05 was considered statistically significant.

## 5. Conclusions

This study highlights key findings regarding age- and sex-related patterns of inhalant allergen sensitization in the Lithuanian population. Sensitization to at least one allergen was observed in more than half of the individuals, reflecting the growing public health concern associated with allergic conditions. Sensitization was most prevalent in children, particularly to tree pollen and dust mites, with rates declining in adulthood and older age. However, grass pollen remained a consistent allergen across all age groups, highlighting its persistent impact despite the decline in sensitization rates with age.

Sex-related differences in sensitization patterns were also evident. Boys were more frequently sensitized than girls in childhood, a trend likely influenced by hormonal and immunological factors. In adulthood, males showed higher sensitization rates to allergens such as tree pollen, grass pollen, and pets, whereas females exhibited lower overall sensitization rates, except in specific subgroups like older women, who were more frequently sensitized to dust mites and pets.

Our results align with global trends in many aspects, particularly in the predominance of sensitization among males in childhood. However, the lower sensitization rates observed in females of reproductive age and older females compared to males diverge slightly from findings in other populations. These differences may be influenced by local environmental, genetic, or hormonal factors unique to Lithuania.

The significance of these findings lies in their potential to shape the future of allergy prevention, diagnosis, and treatment strategies in Lithuania. By providing a detailed overview of sensitization patterns across age and sex groups, this study offers valuable data for healthcare professionals and policymakers working to optimize allergy management tailored to the specific needs of the Lithuanian population.

To build on these findings, future studies should investigate the relationship between sensitization and clinical manifestations of allergic diseases. Importantly, while sensitization represents a necessary step in the allergic disease process, it does not equate to clinically manifest allergy without corresponding symptoms. Thus, our findings should be viewed as descriptive epidemiological data rather than direct estimates of clinical disease prevalence. Accurate identification of sensitization at the molecular level (specific allergen components rather than entire allergen groups or mixes) is essential to improve diagnostic precision and therapeutic approaches.

Longitudinal cohort studies are particularly needed to track sensitization patterns in the same individuals over time. These would help understand how sensitization evolves with changing environmental exposures, hormonal influences, and other modifying factors. Additionally, conducting similar studies across different countries would provide a broader understanding of regional differences and the mechanisms driving allergen sensitization. It is important to integrate clinical data, including patient histories and allergy symptom severity, with sensitization patterns. This approach would help establish correlations between sensitization profiles and allergic disease manifestation, improving personalized diagnostic and treatment strategies.

## Figures and Tables

**Table 1:** Descriptive statistics of the sample and its subgroups.

	Group	Group Size	Median	IQR	Q1	Q3
**Female**	In total	337 (51.22% of total sample size)	26	29	8	37
	Under 18 y/o	138 (40.95% of all females)	6	7	3	10
	18–44 y/o	153 (45.40% of all females)	33	10	27	37
	Over 44 y/o	46 (13.65% of all females)	48	10.25	46	56.25
**Male**	In total	321 (48.78% of total sample size)	15.5	28	5	33
	Under 18 y/o	172 (53.58% of all males)	5	7	3	10
	18–44 y/o	117 (36.45% of all males)	32	10	26	36
	Over 44 y/o	32 (9.97% of all males)	53	11	48	59

**Table 2:** Differences in sensitization rates to inhalant allergens in different subgroups according to age and sex.

Groups	Total	Sensitization to at Least One Inhalant Allergen	*p*-Value
**Children**	310	197 (63.55%)	
Females	138	86 (62.32%)	0.6870
Males	172	111 (64.53%)	
**Adults 18–44 y/o**	270	165 (61.11%)	
Females	153	84 (54.90%)	0.0167
Males	117	81 (69.23%)	
**Older than 44 y/o**	78	47 (60.26%)	
Females	46	25 (54.35%)	0.2011
Males	32	22 (66.75%)	

## Data Availability

Data is contained within the article.
